# Appendix tip biopsy for Total colonic aganglionosis: technical aspects and diagnostic accuracy in a prospective cohort of 107 pediatric patients

**DOI:** 10.1007/s00383-026-06656-4

**Published:** 2026-09-25

**Authors:** Judith Lindert, Laura Stigler, Carla Sternke-Curvello, Andreas Erbersdobler, Stefanie Märzheuser

**Affiliations:** 1 Department of Pediatric Surgery, University Medicine Rostock, Rostock, Germany; 2 Institute of Pathology, University Medicine Rostock, Rostock, Germany

**Keywords:** Total colonic aganglionosis, Appendix biopsy, Hirschsprung disease, Ganglion cells

## Abstract

**Introduction:**

The diagnosis of Total colonic aganglionosis (TCA) remains challenging, frequently resulting in delayed treatment and multiple surgical procedures. Appendix biopsy has been proposed as a diagnostic technique. Concerns exist regarding reliability. This study evaluated the diagnostic accuracy and technical applicability of appendix tip biopsy in children with suspected TCA.

**Methods:**

A prospective observational study was conducted in 107 consecutive children (≤ 15 years) undergoing abdominal surgery with appendix tip biopsy between October 2022 and September 2024. Patients were allocated to three groups: Hirschsprung disease without TCA (*n* = 25), TCA (*n* = 19), and non-Hirschsprung controls (*n* = 63). Histopathological examination assessed the presence or absence of ganglion cells at the tips of the appendix.

**Results:**

Ganglion cells were identified in 24/25 (96%) patients with Hirschsprung disease not involving the entire colon and in 61/63 (96.8%) controls. Ganglion cells were absent in all patients with TCA. Three inconclusive specimens resulted from inadequate sampling or very advanced inflammatory tissue destruction. Appendix tip biopsy achieved a sensitivity of 100%, specificity of 96.6%, positive predictive value of 86.4%, negative predictive value of 100%, and an overall diagnostic accuracy of 97.2%. Assessment was unaffected by patient age, including neonates, and generally possible in appendicitis when adequate full-thickness biopsy was obtained.

**Conclusions:**

Appendix tip biopsy is an accurate and tissue-sparing adjunct for diagnosing total colonic aganglionosis. When combined with appropriate histopathological assessment, it provides rapid diagnostic information while preserving the proximal appendix for potential future reconstructive procedures. These findings support its incorporation into the diagnostic pathway for children with suspected TCA.

## Introduction

Total colonic aganglionosis (TCA) is a rare disease with an incidence of 1:50,000-100,000, which affects only 2–4% of all Hirschsprung (HD) cases. Patients with TCA have a mortality rate of 2–10% due to Hirschsprung’s associated enterocolitis (HAEC), Ileus, failure to thrive, and other comorbidities within the context of this congenital intestinal disorder [1]. Many Patients undergo several interventions until their diagnosis is established [2]. A delayed diagnosis can lead to serious complications such as HAEC, Sepsis, malnutrition, and multiple surgeries [3]. Early diagnosis is crucial to avoid unnecessary interventions, complications, and hospitalization [4].

Due to the rare incidence of TCA, existing studies on the usability of the appendix have been limited by small case numbers over extended periods. While recent studies by Reppucci et al. (2022) [5] and O’Hare et al. (2016) [6] support the reliability of appendix tip biopsy for TCA diagnosis, controversy persists due to earlier case reports describing misdiagnosis when appendix biopsy was used as the sole diagnostic criterion [7, 8]. As a superregional referral centre for pediatric colorectal disorders, we prospectively assembled a large cohort of 19 patients with total colonic aganglionosis (TCA) over 24 months. This study aimed to validate the diagnostic accuracy of appendix tip biopsy in a recent European cohort by systematically assessing ganglion cell (GC) presence across three pediatric groups: patients with TCA, patients with non-TCA Hirschsprung disease, and non-Hirschsprung controls. We report the feasibility and technical approach of an appendix tip biopsy and hypothesised that GC are consistently present in the appendix of non-TCA patients and absent in TCA, supporting appendix tip biopsy as a reliable adjunct diagnostic tool when interpreted by experienced pathologists.

## Methods

**Fig. 1:**
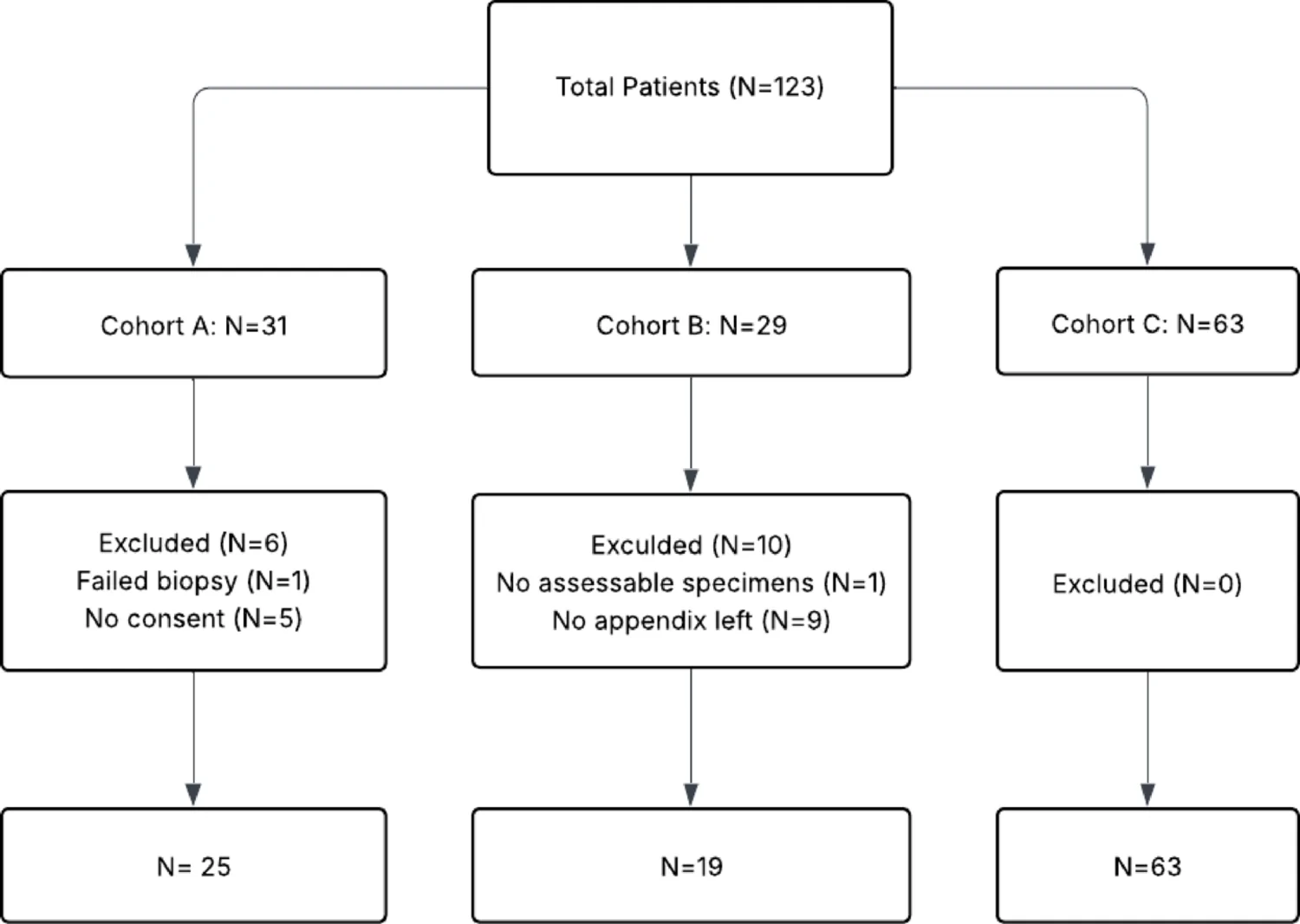
Flowchart of patient inclusion in the different cohorts

A combined prospective, non-randomised single-centre study was designed. Patients undergoing laparoscopy/laparotomy for mapping and /or corrective surgery with their native appendix in situ up to the age of 15 years were included in our study between October 2022 and September 2024 (*N* = 107) (Fig. 1). Consent for the biopsy was obtained from the parents/legal guardian.

**Fig. 2:**
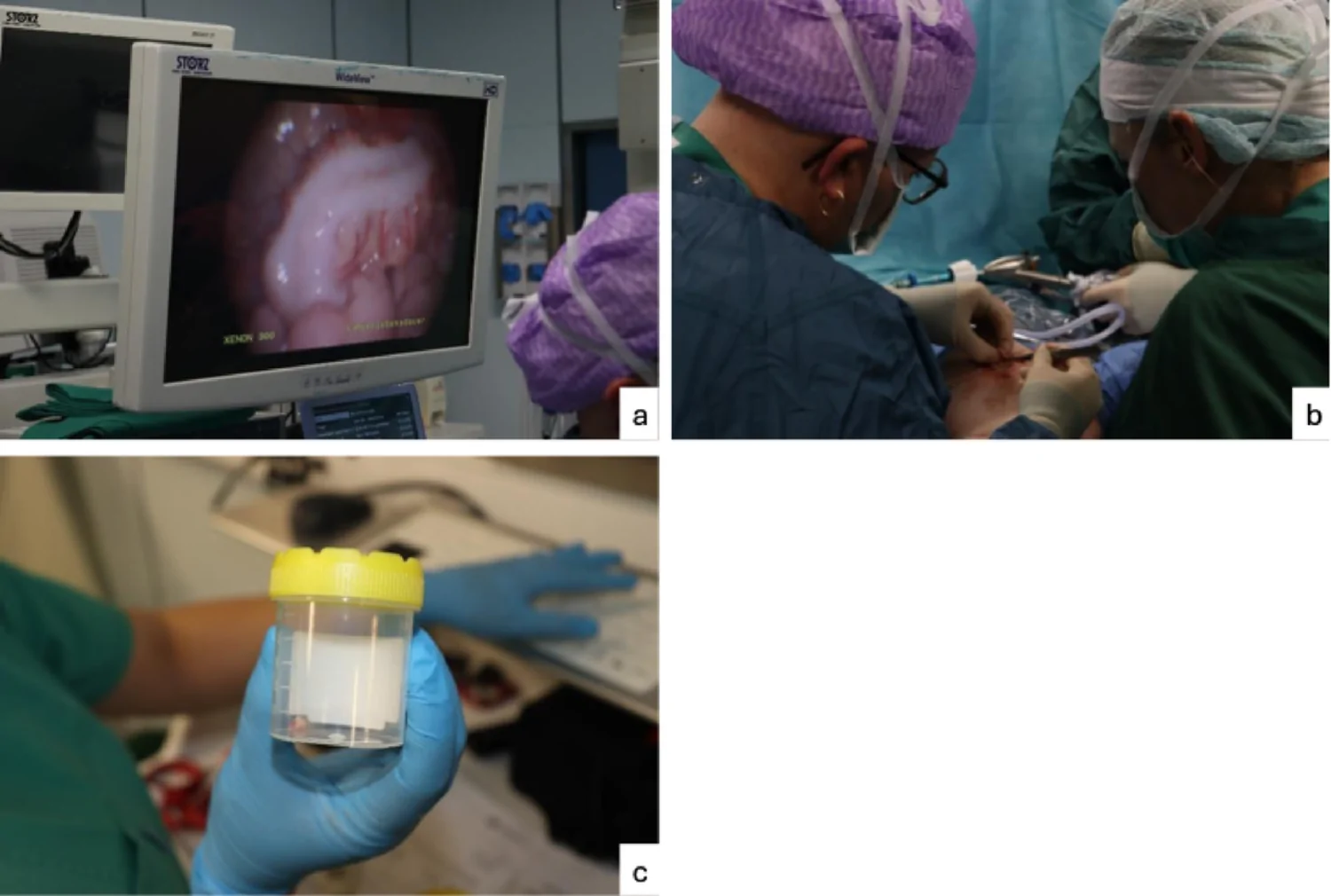
Technical aspects of appendix tip biopsy and histology: **a** laparoscopic identification of the appendix and eventration via the single port, **b** resection of the appendix tip and closing of the remaining appendix with inverted absorbable stitches, **c** appendix specimen before fixation with formalin

Patients were divided into 3 cohorts. Cohort A consisted of all HSCR patients without TCA, Cohort B consisted of TCA patients, and Cohort C consisted of patients without any HSCR variant. In Cohort A, biopsies were predominantly obtained during the pull-through procedure via single-port laparoscopy, which is part of the usual pull-through. In Cohort B, biopsies were obtained during mapping/stoma relocation or the final colectomy and ileoanal pull-through surgery. In two cases, the exact intervention during which the appendix tip biopsy was obtained could not be determined, as it had been performed at a different hospital, though by the same lead surgeon. In Cohort C, biopsies were collected during laparoscopic appendectomy for appendicitis or other intestinal surgery (neonates), and the appendix tip was harvested after resection. Appendix tip biopsies were obtained by resecting 1 cm of the distal appendix using scissors (Fig. 2).

The specimen included a full-thickness wall with both submucosal and muscular layers; for an adequate biopsy, the lumen needed to be visible. The remaining appendix was preserved using an inverted suture with Vicryl 4 − 0. In cases of acute appendicitis requiring complete appendectomy, the distal 1 cm was dissected after removal of the appendix and analysed separately. Most of the specimens were formalin-fixed, stained with haematoxylin and eosin (HE), and studied for the presence of GC in our histopathological institute by pathologists experienced in Hirschsprung disease diagnosis. Ganglion cell examination was performed on frozen sections in two cases.

As this study was based on routine clinical cases, histological examination was performed according to standard clinical practice. In those two cases, a rapid intraoperative assessment was required because the surgical decision depended on the immediate result. Therefore, frozen-section examination was performed in these cases to provide the information needed for the intraoperative decision-making. In those two frozen-section specimens, specimens were sent fresh, and the other normal specimen was sent with fornaling according to institutional protocol. In those two frozen-section specimens, specimens were sent fresh, and the other normal specimen was sent with fornaling according to institutional protocol. We have added this to the methods for clarification.

Statistical analysis was performed using SPSS Statistics 25 and Microsoft Excel. Sensitivity, specificity, positive predictive value (PPV), and negative predictive value (NPV) were calculated with 95% confidence intervals. Statistical comparisons between groups were performed using Fisher’s exact test for categorical variables and the Mann–Whitney U test for continuous variables. P-values < 0.05 were considered statistically significant (See Table 1).

Ethical vote obtained from the University Rostock A 2022 − 0187, 30.11.2022.

## Results

A total of 107 patients undergoing appendix tip biopsy were included (Table 1). Of 123 initially identified patients, 16 were excluded due to insufficient biopsy material (*N* = 2), previous appendectomy (*N* = 9), or missing parental consent (*N* = 5) (Table 2).

**Table 1:** Patient cohort characteristics: HSCR, B: TCA, C: Non-TCA/HSCR

Cohort	Number of patients	Female patients	Age at biopsy (in months)	Weight at biopsy (in kg)	GC detectable
A	25	8	20,7 (4-156)	11.5 (4,2–52)	24
B	19	5	22.6 (3–91)	11.2 (4.1–28.5)	0
C	63	23	98.9 (0–180)	33.1 (1–89)	61
Total	107	36	67,6 (0–180)	24,9 (1–89)	85

**Table 2:** Interventions: A (*N* = 25), B (*N* = 17), C(*N* = 63)

A	Laparoscopic appendectomy	Open appendectomy	Colonmapping	Pull-through (de la Torre)	Re-do pull-through	Stoma surgery	Others	Total
	0	0	1	21	2	1	0	25
B	0	0	0	13	0	4	0	17
C	52	2	0	0	0	5	4	63
Total	52	2	1	34	2	10	4	105

**Fig. 3:**
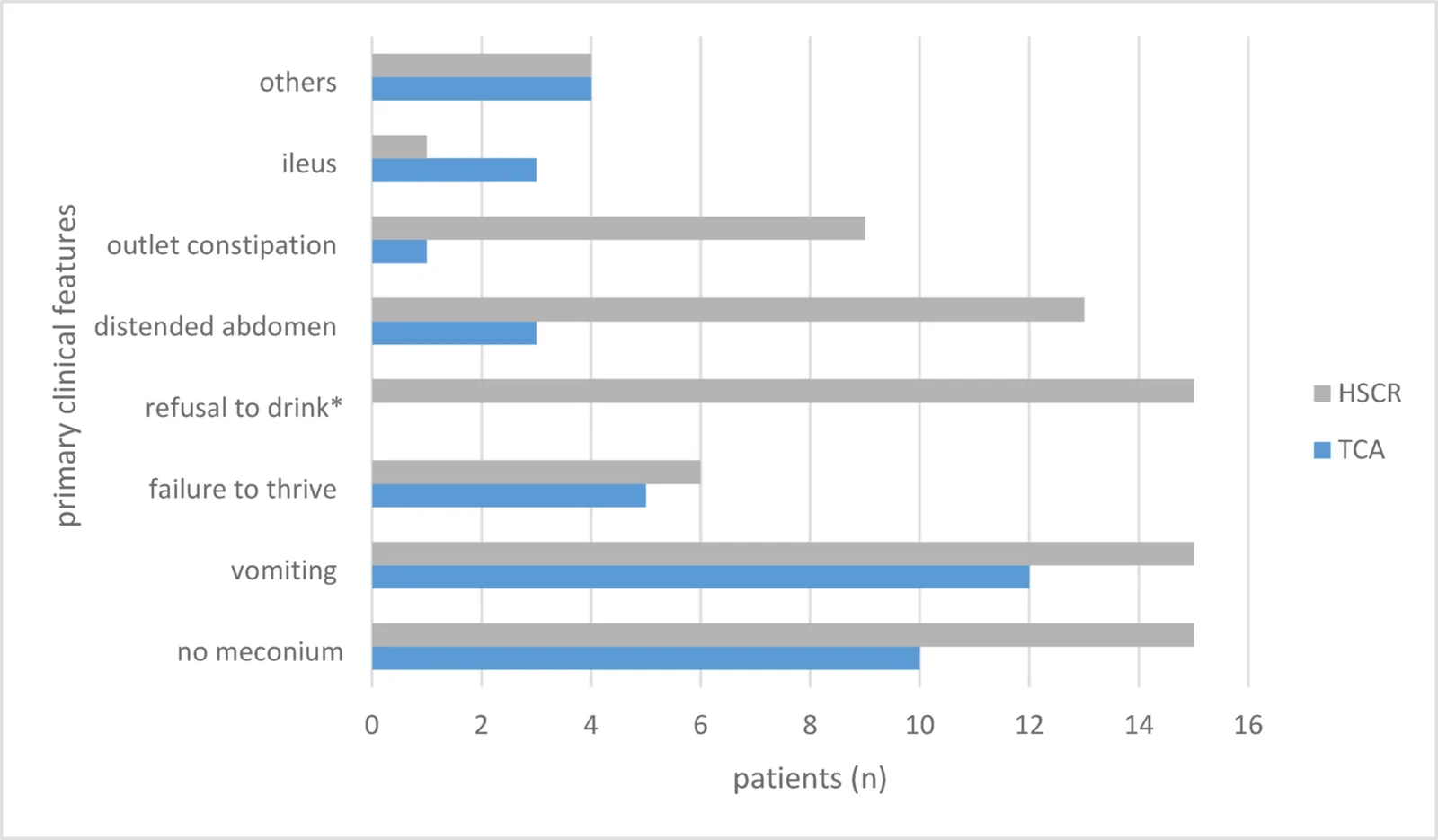
Clinical Presentation of children with Hirschsprung Disease: TCA Patients (*N* = 18, blue) and HSCR patient (*N* = 21, grey), *only assessed in HSCR patients

*Cohort A* (*N* = 25) comprised 8 females (32%) and 17 males, with a median age of 20,7 months (range 4–156) and median weight of 11.5 kg (4.2–52). Seven patients were younger than 6 months. Clinical features prompting suspicion of Hirschsprung disease (HSCR) were documented in 21/25 patients, most commonly delayed passage of meconium within 48 h (62.5%), recurrent vomiting (62.5%), and abdominal distension (54.2%). Refusal to feed occurred in 62.5% (*n* = 15), outlet constipation in 37.5% (*n* = 9), and failure to thrive in 25.0% (*n* = 6); ileus was present in one patient (4.2%) (*n* = 1), while 16.7% (*n* = 4) presented with nonspecific symptoms (Fig. 3). Rectosigmoid disease predominated (68%), followed by long-segment HSCR extending to the left colic flexure (16%).

**Fig. 4:**
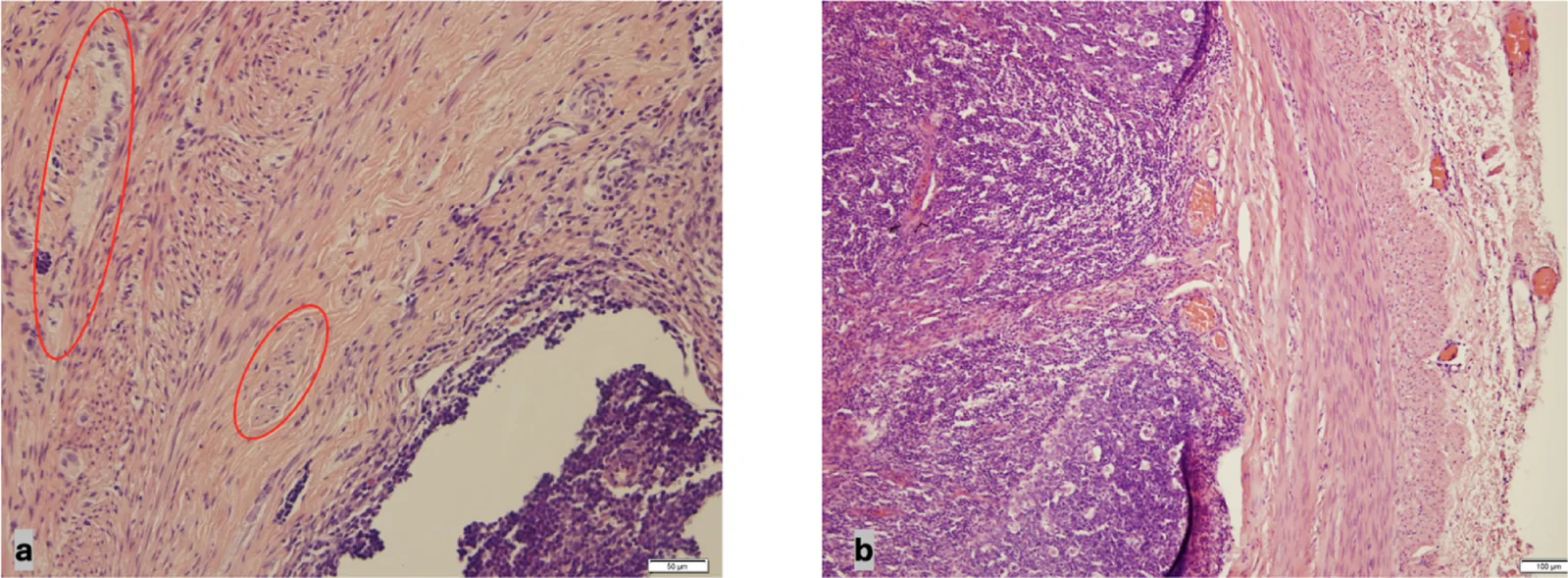
Histopathological assessment for ganglion cells. **a** Ganglia visualised in the Plexus submucosa (red circle) in cohort A (Hirschsprung Disease). **b** No Ganglia visualised in cohort B (TCA)

*Cohort B* (*N* = 19) included 5 females (26.3%) and 14 males, with a median age of 22.6 months (3–91) and median weight of 11.2 kg; four patients were under 6 months of age. Clinical suspicion of total colonic aganglionosis (TCA) was documented in 18/19 patients. Recurrent vomiting in 66.7% (*n* = 12) and delayed meconium passage in 55.6% (*n* = 10) were the most frequent presentations, followed by failure to thrive 27.8% (*n* = 5) and abdominal distension or ileus each 15.8% (n = x). One patient had a inability to open his bowels, and four presented with nonspecific symptoms (Fig. 4). Ileal involvement was present in 14 patients (73.7%), with a mean affected length of 31.5 cm (range 0–96 cm).

*Cohort C* (*N* = 63) consisted of 23 females (36.5%) and 40 males, with a median age of 98.9 months (0–180) and median weight of 33.1 kg. Three patients were younger than 6 months, including two neonates (NEC). Most patients underwent surgery for acute appendicitis (73%, *n* = 46), classified according to European Association for Endoscopic Surgery (EAES) criteria: 63.5% (*n* = 40) were complicated and 4.8% (*n* = 3) uncomplicated appendicitis. Interval appendectomy for chronic-recurrent appendicitis was performed in 10.5% (*n* = 6), with no signs of acute inflammation at surgery. Histopathological classification (Carr, 2000) showed ulcero-phlegmonous appendicitis in 39.7% (*n* = 25), gangrenous appendicitis in 17.5% (*n* = 11), and perforated appendicitis in 7.9% (*n* = 5). Additional indications for appendix tip biopsy included bowel resections and ostomy procedures.

Ganglion cells (GC) were identified in 24/25 specimens (96%) in Cohort A; the single negative result was attributable to inadequate mucosal-only sampling. In Cohort B, 19/19 specimens (100%) demonstrated complete absence of GC. One discordant case initially showed a single GC in the intraoperative frozen section, whereas HE staining of the permanent section confirmed aganglionosis; TCA was subsequently confirmed on multiple colonic and ileal biopsies, without adverse clinical consequences. In Cohort C, GC were detected in 61/63 specimens (96.8%). In two cases with advanced inflammatory destruction, GC could not be reliably identified. See examples of each group in Fig. 4.

**Fig. 5:**
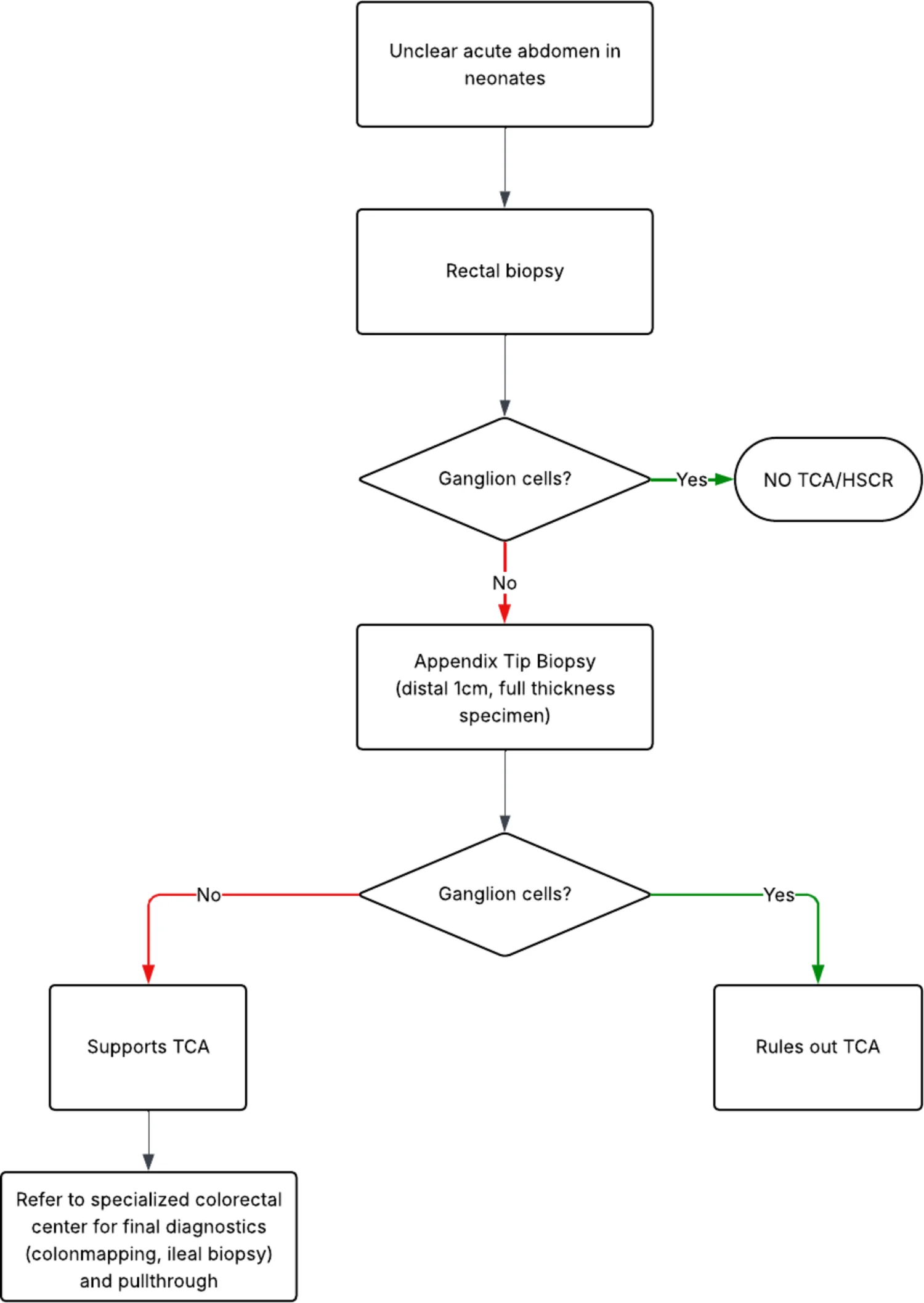
Diagnostic algorithm for total colonic aganglionosis

Appendix tip biopsy demonstrated high diagnostic performance for suspected TCA, with a sensitivity of 100% (95% CI 82.4–100), specificity of 96.6% (95% CI 90.4–99.0), positive predictive value of 86.4% (95% CI 66.7–95.3), and negative predictive value of 100% (95% CI 95,7–100). Overall diagnostic accuracy was 97.2%, with 104/107 patients correctly classified. The positive likelihood ratio was 29.3 and the negative likelihood ratio 0.00. A proposed diagnostic algorithm is shown in Fig. 5.

To evaluate the influence of age on GC detectability, neonatal (≤ 28 days) and non-neonatal groups were compared using Fisher’s exact test. No neonates were included in Cohorts A or B. In Cohort C, GC were detected in both neonatal specimens (*p* = 1.00). A secondary analysis comparing patients ≤ 6 months and > 6 months showed no difference in GC detection rates (76.9% vs. 82.0%; *p* = 0.705). Comparison of correctly classified (*N* = 100) and misclassified patients (*N* = 5) revealed no significant difference in age at biopsy (Mann–Whitney U = 177.0, *p* = 0.272).

There were no complications related to appendiceal suture insufficiency. In children with TCA, the appendix was subsequently removed as part of the colectomy. On macroscopic inspection of the colectomy specimens, the previously biopsied appendices appeared normal. In children with histologically normal appendiceal biopsies, the appendix was left in situ.

At our institution, transanal irrigation is generally preferred for bowel management; consequently, no Malone appendicostomy procedures were performed. Nevertheless, the appendix was preserved in children with normal appendiceal histology, thereby maintaining the option of a Malone procedure at a later stage if required. Similarly, no patient required a Mitrofanoff procedure during follow-up, although this option remains available if clinically indicated.

Follow-up extended beyond the predefined study period, as the children continued to be managed at our institution. At the time of analysis, some patients had been followed for more than four years, allowing assessment of longer-term outcomes.

The available literature of key publications included smaller cohorts of children with Total colonic aganglionisis with a time period up to 10 years; see Table 3.

**Table 3:** Other studies on Appendix Biopsy assessing Ganglion Cells

Study	Time frame	TCA (*N*)	Total (*N*)	Neonates total (*N*)	Sensitivity	Specificity	Method
O’Hare	2006–2016	9	91	–	100%	100%	Permanent
Mohanty	36 months	13	48	30	–	–	Frozen + Permanent
Reppucci	2015–2020	9	112	28	100%	100%	Permanent+Calretinin
Lindert, Stigler-this study	2022–2024	19	107	2	100%	96.6%	Permanent + Frozen

## Discussion

This study demonstrates that an appendix tip biopsy is a reliable diagnostic adjunct for total colonic aganglionosis (TCA), with a sensitivity of 100% and specificity of 96.6% in a cohort of 107 pediatric patients. Our series of 19 recent TCA cases undergoing abdominal surgery (mapping and corrective surgery) represents the largest single-centre cohort reported to date, collected prospectively over 24 months. We are for the first time also comparing with children with Hirschsprung disease not involving the entire colon (regular Hirschsprung disease). Importantly, diagnostic accuracy was maintained across all age groups, including neonates, and was minimally affected by appendiceal inflammation when adequate tissue sampling was achieved. The diagnosis of TCA remains challenging, primarily due to delayed recognition. Patients with TCA reportedly undergo a mean of 6.8 surgical procedures before a definitive diagnosis is established [9], with extended TCA requiring significantly more interventions than short-segment Hirschsprung disease (median 4 vs. 1, *p* = 0.002) [10]. Diagnostic delay is largely attributable to nonspecific clinical presentation, the rare nature of the disease, and limited experience of clinicians not specialised in TCA. Limited sensitivity of contrast enema studies further challenges the establishment of a diagnosis, as a clear transition zone is only visual in 8.8% of cases. Historical series have reported diagnostic delays in up to 37.5% of patients [10, 11], with associated anomalies further obscuring diagnosis [9].

Histopathological examination of serial mapping biopsies remains the diagnostic gold standard for TCA [12]. Contrast enema findings are often nonspecific; features such as microcolon or the “question mark” sign lack diagnostic specificity, and the rectosigmoid index may be falsely normal, particularly in long-segment or total aganglionosis. Contrasting of the small bowel via contrast enema (Ileal influx), frequently observed in neonates, is similarly nonspecific [13]. The rectosigmoid index may appear falsely normal, particularly in cases of long-segment or total aganglionosis. Ileal reflux may be visualized as a nonspecific finding in neonates. Consequently, radiological findings may mimic other causes of neonatal bowel obstruction, increasing the risk of misdiagnosis and inappropriate surgical intervention [13, 14]. Anorectal manometry is not suitable due to technical limitations and the requirement for patient cooperation [15].

The suitability of an appendix biopsy for diagnosing TCA has been debated [5, 6, 8]. Concerns have been raised regarding the identification of immature ganglion cells (GC) in neonates and preterm infants, as GC may appear smaller and less conspicuous [8]. In our cohort, GC were reliably identified in all neonatal and infant samples with adequate tissue quality. Across all three cohorts, 14 patients were younger than 6 months, and GC detectability did not differ compared to older children [6]. These findings challenge previous concerns regarding age-dependent GC immaturity and support the reliability of appendix tip biopsy across all pediatric age groups, including neonates. Our results are consistent with those of Reppucci et al., who demonstrated reliable GC identification in 28 neonatal appendices. Adequate sampling, including muscular layers, and interpretation by experienced pediatric pathologists appear to be the key determinants of diagnostic reliability.

Advanced appendiceal inflammation has been suggested to compromise GC identification due to morphological mimicry by proliferating endothelial cells, lymphocytes, or macrophages, as well as inflammatory distortion of GC themselves [16]. In our study, 46 patients underwent appendectomy for acute appendicitis, predominantly ulcero-phlegmonous or gangrenous. In only two cases did severe inflammatory destruction preclude reliable GC identification. In all remaining cases, GC were consistently identified irrespective of inflammatory severity, and no convincing morphological mimicry by inflammatory cells was observed.

The potential role of adjunctive immunohistochemical staining in cases with extensive tissue destruction warrants further evaluation.

Isolated reports of false-positive appendix biopsies have previously led to recommendations against the use of the appendix as a diagnostic site for TCA. Notably, Lane et al. described a patient with clinical suspicion of TCA in whom appendiceal aganglionosis prompted ileostomy, while subsequent rectal biopsies demonstrated normal innervation [7]. Similar cases have been cited to caution against reliance on appendiceal tissue alone [17].

More recent studies by Reppucci et al. and O’Hare et al., however, have challenged this position, demonstrated high diagnostic accuracy of appendix tip biopsy when appropriately interpreted [5, 6]. Our findings support this evolving evidence base, assessing an even larger number of TCA patients (19 vs. 9) within a shorter period.

In our study, GC were consistently present in both non-TCA cohorts, with exceptions attributable to inadequate sampling (*N* = 1) or severe inflammatory destruction (*N* = 2). In the TCA cohort, all patients demonstrated a complete absence of GC.

Studies from Anderson et al. (1986) and Shaw et al. (1990) reported no differences in the different HSCR subgroups and secured aganglionosis in the appendix at all times [18, 19]. We could not find any differences between short and long-segment HSCR regarding the GC appearance.

The use of the appendix tip biopsy offers clinical advantages. Harvesting only the distal 1 cm provides diagnostic information while preserving the proximal appendix for potential future procedures. Post-pull-through faecal incontinence occurs in up to one-third of patients with Hirschsprung disease [20, 21] and antegrade continence enemas via Malone appendicostomy remain an important management option [22, 23]. Additionally, patients with associated urological anomalies may benefit from a Mitrofanoff appendicovesicostomy. Complete appendectomy performed solely for diagnostic purposes would preclude these options and necessitate more complex alternative conduits.

### Limitations

As a single-centre study conducted in a high-volume tertiary colorectal referral centre, diagnostic performance may not be generalisable to settings with less specialised pathology expertise. Calretinin immunohistochemistry was not routinely employed, which can have improve specificity in equivocal cases. Additional limitations include selection bias towards surgically managed patients, lack of systematic assessment of GC density, unmeasured inter-observer variability, and the partially retrospective study design.

The strength of this study is the prospective collection of a large case cohort, including normal Hirschsprung cases as controls over a short 2-year period.

Future research should focus on prospective, multicentre validation with systematic use of immunohistochemistry, quantitative assessment of GC density and morphology, and correlation with long-term functional outcomes, including bowel function, quality of life, and Hirschsprung-associated enterocolitis.

## Conclusion

In neonates with an unclear intestinal obstruction or patients suggestive of Total Colonic Aganglionisis a properly performed appendix tip biopsy represents a valuable first-line diagnostic adjunct, supplementing the rectal biopsy and avoiding extensive mapping. This study demonstrates high sensitivity and specificity across all pediatric age groups, including neonates, challenging previous concerns regarding GC identification in young infants. Reliable diagnosis depends on adequate tissue sampling, including muscular layers, and interpretation by experienced pathologists. By preserving the proximal appendix, this technique maintains future surgical options while providing timely diagnostic information. When integrated into a comprehensive diagnostic algorithm alongside clinical assessment and confirmatory rectal biopsies, appendix tip biopsy represents a safe, effective, and tissue-sparing tool in the evaluation of suspected TCA.

## Data Availability

No datasets were generated or analysed during the current study.
